# HER2 amplification subtype intrahepatic cholangiocarcinoma exhibits high mutation burden and T cell exhaustion microenvironment

**DOI:** 10.1007/s00432-024-05894-0

**Published:** 2024-08-28

**Authors:** Xiaohong Pu, Lin Li, Feng Xu, Ziyu Wang, Yao Fu, Hongyan Wu, Jun Ren, Jun Chen, Beicheng Sun

**Affiliations:** 1 Department of Pathology, Nanjing Drum Tower Hospital, The Affiliated Drum Tower Hospital of Medical School, Nanjing University, Nanjing, 210008 Jiangsu Province China; 2 Department of Medical Imaging, The Affiliated Suqian First People’s Hospital of Nanjing Medical University, 223800, Suqian, Jiangsu Province China; 3 Department of General Surgery, Northern Jiangsu People’s Hospital, Yangzhou University, Yangzhou, 225000 Jiangsu Province China; 4 Medical School, Nanjing Drum Tower Hospital, The Affiliated Drum Tower Hospital of Medical School, Nanjing University, Nanjing, 210008 Jiangsu Province China

**Keywords:** Intrahepatic cholangiocarcinoma, HER2 status, In fluorescence in situ hybridization, Immunohistochemistry, T cell infiltration

## Abstract

**Objective:**

This study aimed to establish a uniform standard for the interpretation of HER2 gene and protein statuses in intrahepatic cholangiocarcinoma (ICC). We also intended to explore the clinical pathological characteristics, molecular features, RNA expression and immune microenvironment of HER2-positive ICC.

**Methods:**

We analyzed a cohort of 304 ICCs using immunohistochemistry (IHC) and fluorescence in situ hybridization (FISH) to identify HER2 status. Comprehensive analyses of the clinicopathological, molecular genetic, and RNA expression characterizations of ICCs with varying HER2 statuses were performed using next-generation sequencing. We further investigated the tumor microenvironment of ICCs with different HER2 statuses using IHC and multiplex immunofluorescence staining.

**Results:**

HER2/CEP17 ratio of ≥ 2.0 and HER2 copy number ≥ 4.0; or HER2 copy number ≥ 6.0 were setup as FISH positive criteria. Based on this criterion, 13 (4.27%, 13/304) samples were classified as having HER2 amplification. The agreement between FISH and IHC results in ICC was poor. HER2-amplified cases demonstrated a higher tumor mutational burden compared to non-amplified cases. No significant differences were observed in immune markers between the two groups. However, an increased density of CD8 + CTLA4 + and CD8 + FOXP3 + cells was identified in HER2 gene-amplified cases.

**Conclusion:**

FISH proves to be more appropriate as the gold standard for HER2 evaluation in ICC. HER2 gene-amplified ICCs exhibit poorer prognosis, higher mutational burden, and T cell exhaustion and immune suppressed microenvironment.

**Supplementary Information:**

The online version contains supplementary material available at 10.1007/s00432-024-05894-0.

## Background

Intrahepatic cholangiocarcinoma (ICC) is the second most prevalent hepatic malignancy, surpassed only by hepatocellular carcinoma (HCC) (Gupta and Dixon 2017). However, ICC exhibits a more aggressive biological behavior and carries a worse prognosis than HCC (Xue et al. 2019). Despite a global increase in ICC incidence over recent decades, therapeutic options presently available are limited (Braconi and Patel 2010). Over the past few decades, numerous studies have focused on investigating the genomic landscape of intrahepatic cholangiocarcinoma (ICC), revealing the presence of key oncogenic drivers that may serve as potential targets for therapeutic interventions. Recurrent alterations in *IDH1* and *FGFR2* genes have been observed almost exclusively in ICC patients compared to those with extrahepatic cholangiocarcinoma (Lowery et al. 2018; Churi et al. 2014; Jain and Javle 2016). In fact, the United States has approved drugs such as pemigatinib and ivosidenib for the treatment of cholangiocarcinoma patients with *FGFR2* fusions/rearrangements and *IDH1* mutations. Genomic analysis of cholangiocarcinoma patients has also identified other targetable oncogenes in approximately 50% of cases (Lowery et al. 2018). One of the extensively studied biomarkers is the human epidermal growth factor receptor 2 (*HER2*), which plays a pivotal role in tumor proliferation through downstream signaling activation (Yarden and Sliwkowski 2001). HER2 amplification/overexpression has been recognized as a predictive biomarker for HER2-targeted agents in various tumor types, including breast and gastric cancers. Although HER2-targeted therapy has shown improved clinical outcomes in HER2-positive breast and gastric cancer patients (Slamon et al. 2001; Bang et al. 2010; Oh and Bang 2020; Olson 2012), limited data is available regarding the frequency of ICCs with HER2-positive status and the efficacy of HER2/neu targeted therapy in ICCs. However, several case reports have explored the potential of HER2-targeted therapy in biliary tract cancers (BTCs) (Bang et al. 2017; Javle et al. 2015; Nam et al. 2016), demonstrating objective response rates of 64% and 22% in HER2-positive BTC patients treated with HER2-targeted therapies in two phase II studies (Hainsworth et al. 2018; Hyman et al. 2018). In addition to HER2 amplification/overexpression, there is a subset of HER2-negative tumors that express low levels of HER2 protein (HER2-low) detected by immunohistochemistry (IHC), i. e., at 1 + or 2 + intensity, and lack *HER2* amplification by in situ hybridization techniques. Although HER2-targeted therapies are not effective in patients with HER2-low-expressing tumors (Fehrenbacher et al. 2020), recent studies have shown promising therapeutic activity using HER2-directed antibody–drug conjugates (ADCs) with chemotherapeutics, such as trastuzumab deruxtecan (T-DXd) and trastuzumab duocarmazine (SYD985), in HER2-low breast cancer patients (Rinnerthaler et al. 2019; Modi et al. 2020). The potential of ADCs in the treatment of ICC is also encouraging. Overall, these findings underline the significance of understanding the genomic landscape of ICC and the potential of targeted therapies, particularly those focused on HER2, in improving the clinical outcomes of patients with ICC.

Accurate diagnosis is crucial for precise treatment planning. However, the criteria for determining HER2 amplification/overexpression in intrahepatic cholangiocarcinoma (ICC) are not well-established, as they primarily rely on criteria developed for breast and gastric cancers (Schalper et al. 2014). Furthermore, *HER2* mutations have been identified in ICC, and early results indicate their potential as targets for treatment. Nevertheless, the clinicopathological characteristics associated with HER2 amplification/overexpression or mutation subtypes in ICC remain poorly understood. Therefore, our study aims to address these gaps. We utilized fluorescence in situ hybridization (FISH) and immunohistochemistry (IHC) techniques to assess the genetic and protein status of HER2 in a comprehensive sample cohort of 304 ICC cases. Additionally, whole exome sequencing (WES) was employed to detect HER2 mutation status. Moreover, a comparison of the immune status between HER2 amplified and non-amplified samples was performed, aiming to explore the potential utility of immunotherapy in the treatment of intrahepatic cholangiocarcinoma (ICC). By doing so, we evaluated the concordance between FISH and IHC results, analyzed the clinicopathological features, immune microenvironment, and gene expression profiles of HER2 amplified/overexpressedICC subtypes.

## Materials and methods

### Patients and samples

**Fig. 1:**
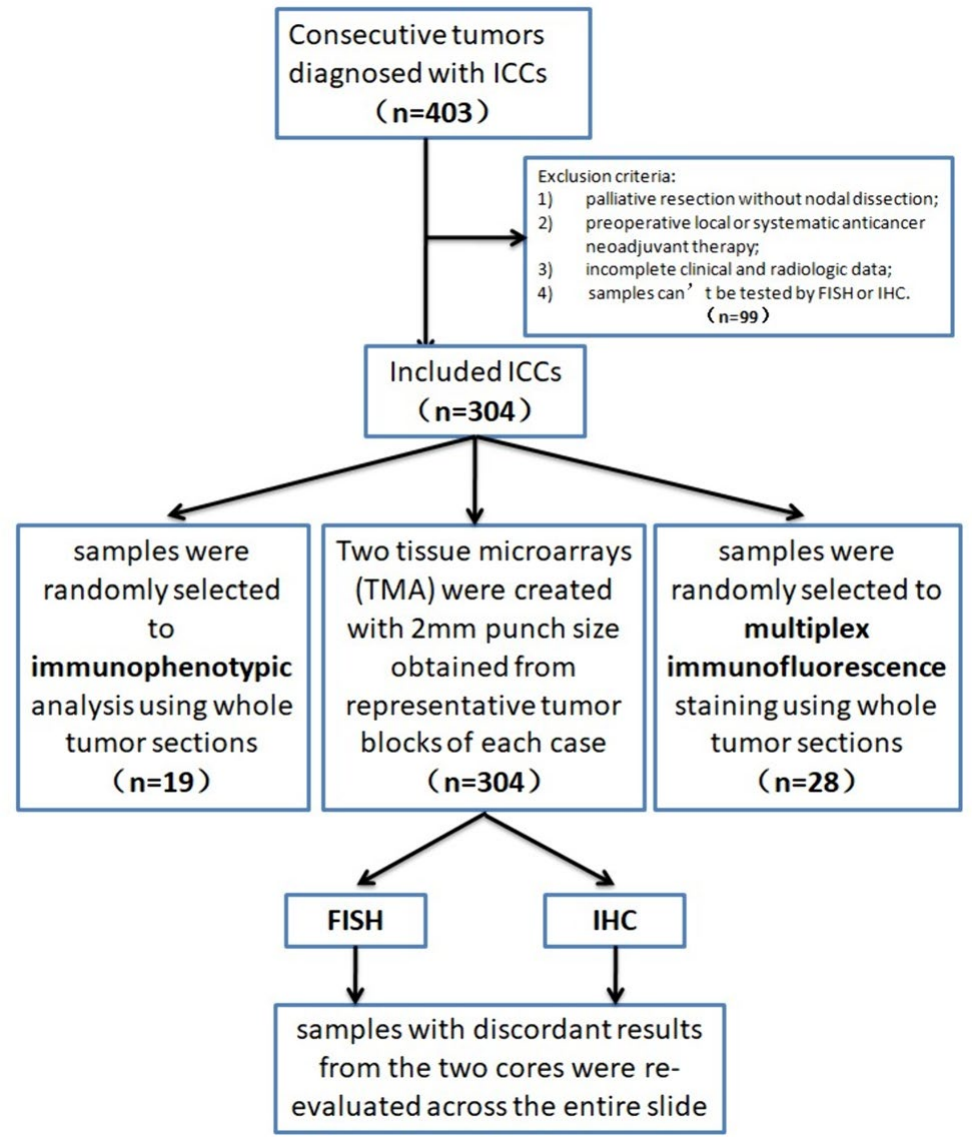
Flowchart of study design

We searched the electronic pathology database in the Department of Pathology of the Nanjing Drum Tower Hospital in China over the period from April 2008 to May 2022 and identified a total of 403 consecutive tumors diagnosed with ICCs in radical resections **(**Fig. 1**)**. The inclusion criteria were as follows: (1) pathologically diagnosed adenocarcinoma, mucinous adenocarcinoma according to the latest WHO classification; (2) radical surgical resection with nodal dissection; (3) complete clinical, radiological and pathological data. Exclusion criteria included: (1) palliative resection without nodal dissection; (2) preoperative local or systematic anticancer neoadjuvant therapy; (3) incomplete clinical and radiologic data; (4) samples can’t be tested by FISH or IHC. Finally, 304 cases were included in this study. Medical records of all patients were reviewed. Tabulated was preoperative information, including the general information, HBV infection, dipsomania, hepatic steatosis, biliary hamartoma, clonorchiasis. The pathological data, such as tumor number, maximum dimension, histologicalclassification, mVI, differentiation (L, low differentiated; M, moderately differentiated; H, high differentiated; U, undifferentiated), and G (grade)/S (stage)/T (tumor) stage were recorded. Tumor TNM staging was determined according to the eighth edition of the American Joint Committee on Cancer (AJCC)/Union for International Cancer Control (UICC) TNM Classification and Stage Groups for ICC. Moreover, because of the scattered large geographic location of the patients and long follow-up duration, only 60.2% (183/304) of patients in researches were obtained the follow-up information. This study was supported by Nanjing Drum Tower Hospital ethics committee.

### Tissue microarray construction

Each tissue sample underwent immediate fixation in 10% neutral buffered formalin for duration of 12–48 h, followed by paraffin embedding. The processed samples were subjected to routine deparaffinization and rehydration procedures. A tissue microarray (TMA) was created using the Grand Master automated array (3DHISTECH Ltd., Budapest, Hungary), with 2 mm punch size obtained from representative tumor blocks of each case. The tumor core was extracted from the invasive front of the deepest tumor invasion portion, with avoidance of necrotic areas. Before constructing tissue microarrays, each sample was subjected to hematoxylin and eosin (HE) staining, and the tumor areas were circled. Subsequently, two cores were randomly selected from the circled tumor areas to create tissue microarrays. ual representations of TMAs were constructed and then sectioned into 4-μm-thick sections for histological, immunohistochemical, and FISH detection procedures. To address the issue of heterogeneity in tissue microarrays, samples with discordant results from the two cores were re-evaluated across the entire slide. This was done to ensure that the selected cores could represent the overall status of the entire tissue section.

### Immunohistochemical staining and analysis

The anti-HER2/neu monoclonal antibody (CST, cot:CST4290) was used for HER2 immunohistochemistry (IHC) staining through an automated protocol validated on the Ventana Bench Mark Ultra system (Roche Diagnostics, Basel, Switzerland). Two pathologists independently interpreted the HER2 IHC results in the absence of clinical or pathological information, and any disagreements were adjudicated through consultation. In cases of inconsistent duplication, the entire slide was stained for final interpretation. HER2 scores were determined by multiplying the positive intensity with the positive percentage. Intensity was scored as follows: 0 = no staining; 1 +  = weak, incomplete membrane staining in > 10% of tumor cells; 2 +  = weak to moderatelycomplete membrane staining in > 10% of tumor cells; 3 +  = strong, complete membrane staining in > 30% of tumor cells. Finally, based on the HER2 scores, we adopted the American Society of Clinical Oncology/College of American Pathologists (ASCO/CAP) recommended criteria (Schalper et al. 2014) for breast cancer and categorized IHC values into 1 + for 1–50, 2 + for 51–150, and 3 + for values greater than 150.

In addition to HER2 evaluation, immunophenotypic analysis of tumor cells and tumor-infiltrating lymphocytes (TILs) was performed using IHC staining for CD20 (Abcam, cot:Ab78237), CD3 (Abcam, cot:Ab135372), CD68 (CST, cot:CST76437), CD8 (CST, cot:CST30706), CD4 (ZSGB-BIO, cot:ZM-0418), and CD163 (ZSGB-BIO, cot:ZM-0428), FOXP3 (Biolegend, cot:BLG320202), and PD1 (ZSGB-BIO, cot:ZM-0381) in whole slide of 19 cases. The IHC scores for CD20, CD3, CD68, CD8, CD4, and CD163 were calculated separately based on the percentage of positive cells in the tumor center and tumor periphery. On the other hand, the percentage of FOXP3 or PD1-positive cells was calculated considering both the tumor center and tumor periphery. 19 samples were randomly selected to immunophenotypic analysis using whole tumor sections. Among these, 7 samples exhibited HER2 amplification, while 12 samples with no HER2 amplification as controls.

### Multiplex immunofluorescence staining and evaluation

Multiplex immunofluorescence staining was performed using the Opal 5-Color Manual IHC Kits (Panovue Biotechnology) following the manufacturer's instructions. Two panels, consisting of CD20 (Abcam, cot:Ab78237), CD3 (Abcam, cot:Ab135372), CD68 (CST, cot:CST76437), HER2 (CST, cot:CST4290), and CK (Sigma, cot:C2562), as well as CK, PD-L1 (CST, cot:CST13684), CD8 (CST, cot:CST30706), FOXP3 (Biolegend, cot:BLG320202), and CTLA4 (Abcam, cot:ab237712), were visualized using the Olyvia System and analyzed with Qupath software (Bainuo, China). The Olyvia System quantitatively assessed the average density (cells/mm2)of each lymphocyte subset or merged lymphocyte subsets across the entire slide. The expression of PD-L1 in tumor-infiltrating lymphocytes (TILs) was evaluated using an immunoreactivity scoring system (IRS). PD-L1 staining was considered positive if any perceptible membrane staining, whether partial or complete, of any intensity distinct from cytoplasmic staining was present. The intensity of staining was categorized as weak (score 1), moderate (score 2), or strong (score 3). PD-L1 expression immunoreactive score (IRS) was calculated by multiplying the positive intensity by the positive percentage. All multiplex immunofluorescence staining was conducted on whole slides. In order to evaluate different HER2 statuses in cholangiocarcinoma samples, a total of 28 cases were randomly included in the multiplex immunofluorescence staining analysis, comprising 11 samples with HER2 amplification and 17 samples without HER2 amplification.

### Fluorescence in situ hybridization

For fluorescent in situ hybridization (FISH), a commercially available, locus-specific HER2 probe and CEP17 probe were used as recommended by the manufacturer (Kanglu, Wuhan, China). At least 20 non-overlapping nuclei of tumor cells per sample were evaluated for HER2 probe (red) and CEP17 probe (green) signals, and the signal counting results of HER2 and CEP17 were recorded for further evaluation. Similar to IHC, FISH was performed in both duplicates of each case, and in case of disagreement between the duplicates in FISH results, the entire slide was used for a final decision.

### Concordance index between IHC and FISH

We used the FISH results as the final outcome to assess the concordance between IHC and FISH results.

### Next-generation sequencing

Out of the 304 cases of ICCs included in the study, 283 cases underwent Whole-Exome Sequencing (WES) analysis. DNA and RNA from tumor tissues were extracted, and sequencing libraries were prepared. DNA-based target sequencing was performed on a panel of 1021 cancer-related genes. Complete DNA and RNA sequencingwas performed on a Gene + Seq2000 (Beijing Gene Plus, Beijing, China.) or DNBSEQ-T7 (Beijing Genomics Institute, Beijing, China.) instrument. Sequencing data were analyzed using the default parameters. Adaptor sequences and low-quality reads were removed before aligning to the reference human genome (hg19) using the Burrows‒Wheeler Aligner (BWA; version 0.7.12-r1039). Realignment and recalibration were performed by using GATK (version 3.4-46-gbc02625). Single nucleotide variants (SNVs) were called using MuTect (version 1.1.4) and NChot, software developed in-house to review hotspot variants. Small insertions and deletions (Indel) were determined using GATK. Somatic copy number variations (CNVs) were identified using CONTRA (v2.0.8). The fusion genes were identified with the NCsv program (in-house) using split reads, discordant pair reads, and single unmapped reads in the alignment file. The final candidate variants were all manually verified using the Integrative Genomics Viewer.

In each group, the frequency of gene mutations was calculated, retaining only genes with a mutation frequency of 10% or higher. Tumor mutation burden was defined as the sum of synonymous and non-synonymous mutations. A waterfall plot was generated using the complexHeatmap function in R (v 4.2.2).

The Wilcoxon signed-rank sum test was employed to assess the differences in gene expression between the two groups. Differentially expressed genes were identified based on a criteria of p value < 0.05 and absolute log2 fold change > 1. Expression profiles of the selected differentially expressed genes were extracted from the original expression data. The data was first normalized by samples, followed by normalization by genes. Subsequently, a heatmap was generated using the pheatmap function in R (v 4.2.2), with sample clustering based on the default parameters of pheatmap.

### Statistical analysis

Overall survival (OS) time was defined as the time in months from surgery to death or the last available follow-up. Patients who were alive at follow-up were censored. Statistical analyses were conducted using IBM SPSS Statistics for Windows, version 23.0. Categorical variables were analyzed using the Wilcoxon rank-sum, chi-square or Fisher's exact test, while continuous variables were analyzed using the *t* test. OS was analyzed using the Kaplan–Meier method and p value relates to the log rank analysis. Univariate analysis was performed for prognostic factors using the log-rank test. All graphs were generated using GraphPad Prism version 9 (GraphPad Software Inc., San Diego, CA). A p value of less than 0.05 was considered statistically significant.

## Results

### Establishing thresholds for HER2 amplification in ICC

**Fig. 2:**
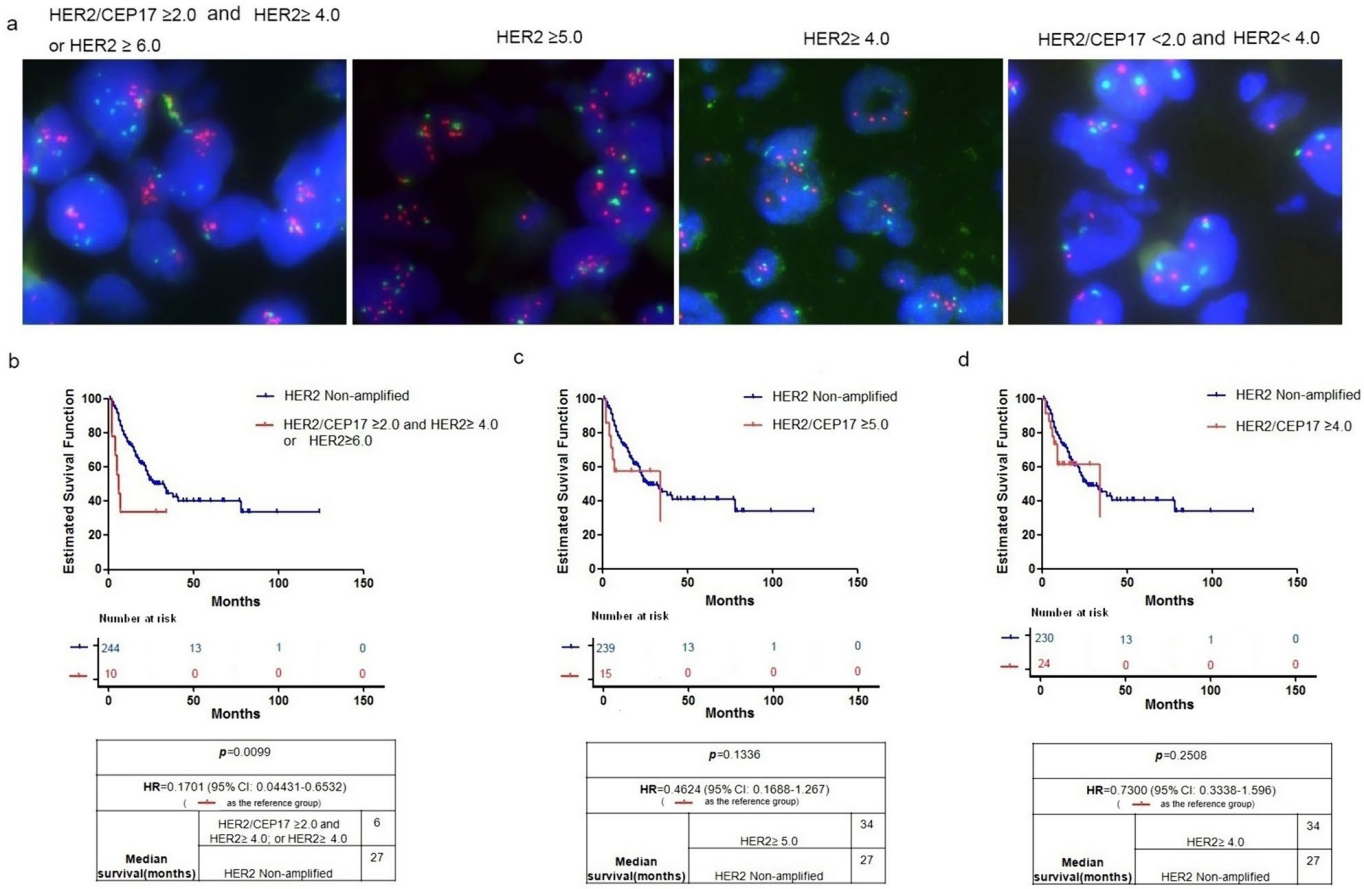
HER2 evaluation by fluorescence in situ hybridization (FISH) in TMA of 304 ICCs. Representative FISH pattern of tumor cells HER2/CEP17 ≥ 2. 0 and HER2 ≥ 4. 0, or HER2 ≥ 6.0; HER2 ≥ 5.0; HER2 ≥ 4.0; HER2/CEP17 < 2.0 and HER2 < 4.0 (**a**). Kaplan–Meier curves of overall survival among FISH subgroups. HER2/CEP17 < 2. 0 and HER2 < 4.0 (HER2 non-amplified) vs. HER2/CEP17 ≥ 2.0 and HER2 ≥ 4.0; or HER2 ≥ 6.0 (**b**), HER2/CEP17 < 2.0 and HER2 < 4.0 (HER2 non-amplified) vs. HER2 ≥ 5.0 (**c**), HER2/CEP17 < 2.0 and HER2 < 4.0 (HER2 non-amplified) vs. HER2 ≥ 4.0 (**d**). p value relates to the log rank analysis and median survival in months for each group was presented in the table

Currently, the definition of HER2 amplification or overexpression in ICC lacks a standardized criterion. To address this issue, we adopted the HER2 amplification criteria for breast (Wolff et al. 2018) and gastric cancer (Cutsem et al. 2015) as reference and established three FISH positive criteria: (1) HER2/CEP17 ≥ 2.0 and HER2 ≥ 4.0; or HER2 ≥ 6.0; (2) HER2 ≥ 5.0; (3) HER2 ≥ 4.0 (Fig. 2a). The established HER2 amplification rates for the three criteria were 4.28% (13/304), 5. 58% (20/304), and 9. 87% (30/304) respectively. We evaluated the values of different HER2 amplification criteria in distinguishing between amplified and non-amplified groups based on clinicopathological features and prognostic outcomes. The results demonstrate that, regardless of the applied criteria, no significant differences were observed in the clinical and pathological features between the amplified and non-amplified groups (Supplemental Table 1). Additionally, we carried out a prognostic analysis among the three different positive standards and found no significant statistical differences in the prognosis among the three groups (Supplemental Fig. 1a). However, we observe a significant statistical difference in overall survival (OS) only when FISH positive criteria specified HER2/CEP17 ≥ 2.0 and HER2 ≥ 4.0; or HER2 ≥ 6.0 (p = 0.0099) (Fig. 2b–d). Thus, we defined HER2 amplification in ICC as HER2/CEP17 ≥ 2.0 and HER2 ≥ 4.0; or HER2 mean value ≥ 6.0 in this study, which is also the interpretation criteria for HER2 fluorescence in situ hybridization (FISH) in breast cancer.

### Establishing thresholds for HER2 IHC and analyzing the consistency between IHC and FISH

**Fig. 3:**
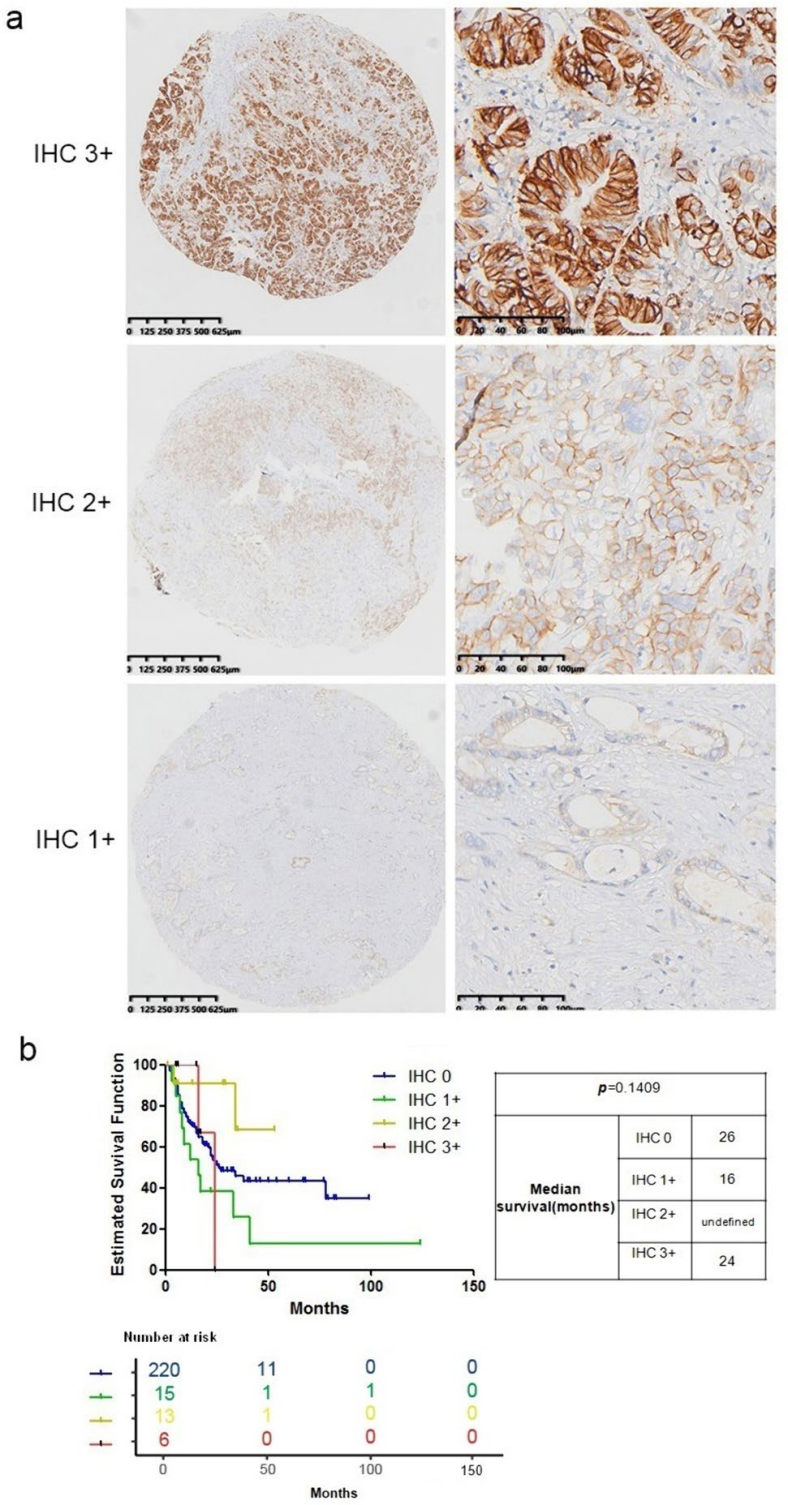
HER2 evaluation by immunohistochemistry (IHC) in TMA of 304 ICCs. Representative immunostaining pattern and intensity of tumor cells: circumferential staining/strong (IHC 3 +), lateral and basolateral staining/moderate (IHC 2 +), lateral or basolateral staining/weak (IHC 1 +) (**a**). Kaplan–Meier curves of overall survival among IHC subgroups (**b**). p value relates to the log rank analysis and median survival in months for each group was presented in the table

Following the establishment of HER2 amplification threshold in ICC, we investigated the concordance between HER2 IHC and FISH. In this study, the overall IHC membranous positivity rate was 14.47% (44/304). Of the 260 cases with an IHC value of 0, eight were FISH positive, while all 18 cases with an IHC value between 1 and 50 were FISH negative. Of the 14 cases with an IHC value between 51 and 150, three were FISH positive, and of the 12 cases with an IHC value between 151 and 300, two were FISH positive (Fig. 3a). Having previously evaluated the value of HER2 gene status in distinguishing between clinicopathological features and prognostic outcomes, we found that only HER2 FISH positive criteria specified HER2/CEP17 ≥ 2.0 and HER2 ≥ 4.0; or HER2 ≥ 6.0 showed a suggestive value in prognostic outcomes. We then investigated whether HER2 protein status could better distinguish between overexpression and non-overexpression groups in terms of clinical and prognostic features. We classified HER2 protein status into four groups based on the results of IHC: IHC 0, 1 + , 2 + , and 3 + but found HER2 protein status are not significantly related to clinicopathological features of ICC (Fig. 3b, Supplemental Fig. 1b–d and Supplemental Table 2).

The absence of IHC membranous positivity did not necessarily imply FISH negativity, with 3.08% (8/260) of the IHC negative cases demonstrating FISH positivity. Moreover, only 16.7% (2/12) of the cases with IHC 3 + showed HER2 amplification (Supplemental Table 3).

### The heterogeneity of HER2 FISH and IHC

A tissue microarray (TMA) was created, and two cores were extracted from each tumor sample, which were subjected to HER2 FISH and IHC analyses. Out of 304 samples analyzed, 23 exhibited inconsistencies in their IHC results between the two cores obtained from the same sample, while FISH showed a much lower degree of heterogeneity compared to IHC. Specifically, only six samples (1.97%, 6/304) were found to exhibit inconsistencies between the two cores, and among these six samples, four were ultimately determined to be HER2-positive. This suggests that the use of puncture samples for IHC testing may potentially influence the use of targeted therapies in patients. More detailed information on the inconsistencies observed between the FISH results from the two core biopsies is provided in (Supplemental Table 4).

### Assessment of HER2 status: gene as the gold standard

Given that immunohistochemistry (IHC) has no informative value in distinguishing clinical and pathological characteristics while exhibiting notable intratumoral heterogeneity, so regarding the assessment of HER2 status, FISH is more appropriate than IHC to serve as the gold standard. And we set HER2/CEP17 ≥ 2.0 and HER2 ≥ 4.0; or HER2 ≥ 6.0 as FISH positive criteria, or HER2 amplified criteria.

### Clinicopathological characteristics of HER2 amplification cases

The ages of patients with HER2 amplification ranged from 52 to 76 years, with a median age of 64.85 years. The incidence rate between genders is nearly equivalent, with seven male patients and six female patients among a total of thirteen individuals. Four patients (5/13) showed HBsAg positivity. According to AJCC TNM staging (8th Edition), seven patients (7/13) were classified as stage I/II, and six patients (6/13) were classified as stage III/IV (Supplemental Table 1). As of the time of this report, a total of seven fatalities among the patient population have been recorded, the overall average survival time was only 14.22 months.

### Somatic aberrations between HER2 amplified and non-amplified ICCs

**Fig. 4:**
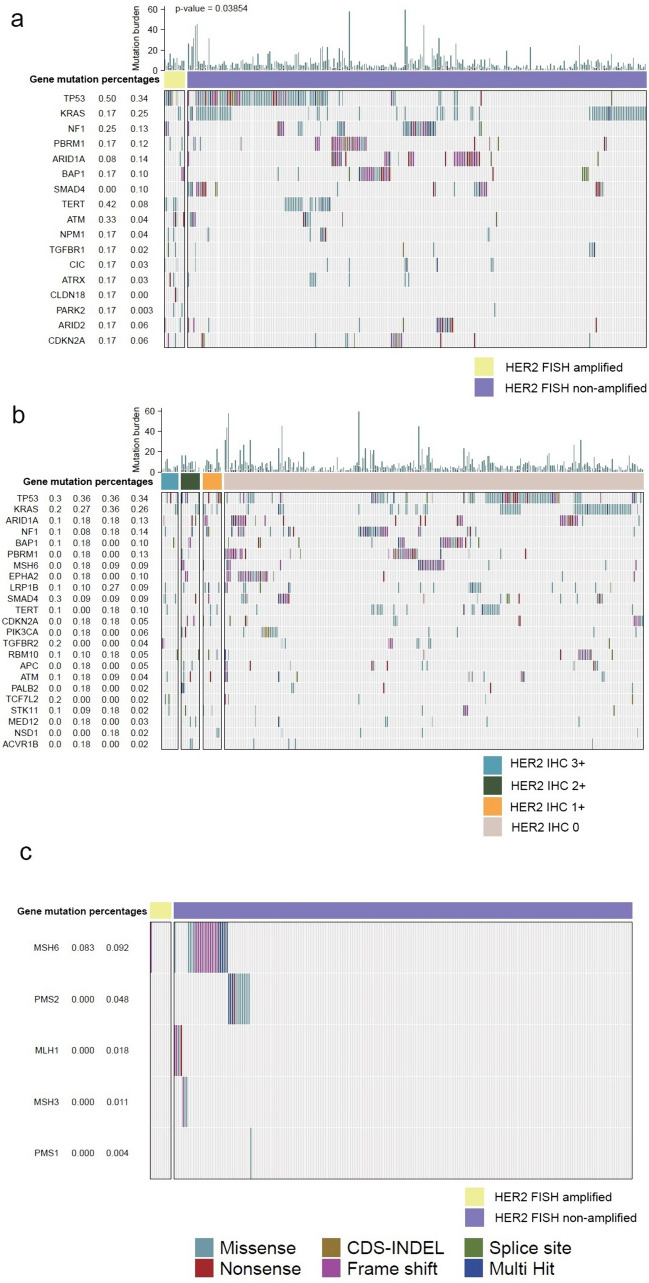
Somatic aberrations between HER2 amplified and non-amplified ICCs (**a**), and somatic aberrations among different HER2 IHC groups (**b**). The mutations of mismatch repair genes between HER2 amplified and non-amplified ICCs (**c**)

Of the 304 Intrahepatic Cholangiocarcinoma (ICC) samples analyzed, 283 underwent sequencing based on DNA. This prophylactic of sequencing encompasses 12 samples manifesting HER2 amplification and 271 samples functioning as controls. Classification of mutations was divided into somatic synonymous and non-synonymous mutations, inclusive of single-nucleotide mutations, small insertions or deletions, InDels, frame-shift mutations, splice site anomalies, and multi-hit alterations. Top ranking among the five genes experiencing the highest mutation frequency within the HER2 amplified faction were TP53 (50%), TER2 (42%), ATM (33%), NPM1 (24%), and NF1 (21%). The non-HER2 amplified grouping displayed an alternate mutation pattern, with the dominant five mutated genes revealed as TP53 (34%), KRAS (25%), ARID1A (14%), NPM1 (13%), and PBRM1 (12%). The mutation burden witnessed a significant decline in the non-HER2 amplified group relative to its HER2 amplified parallel (p = 0.03854) (Fig. 4a). Concurrently, we scrutinized samples from distinct HER2 IHC groups pursuant to next-generation sequencing results, with intriguing outcomes: no substantial variation was noted either in mutational pattern or mutation burden across the different HER2 IHC groups (Fig. 4b). Within the 304 ICC samples, two exhibited deficiencies in mismatch repair proteins, both revealing MLH1 concurrent with PMS2 deficiency and both corresponding to HER2 non-amplified samples. Moreover, while inspecting mRNA expression across four mismatch-repair proteins, we further compared mutation status of mismatch repair proteins, including MLH1, MLH3, MSH3, MSH6, PMS1 and PMS2, between the HER2 amplified and non-amplified groups, illuminating a lower frequency of mismatch repair protein anomalies in the HER2 amplified group.Only one out of 12 HER2 amplified samples carried a mutation in MSH6 (Fig. 4c). Through second-generation sequencing analysis, we also detected 3 cases of HER2 point mutations among the 283 samples. They were HER2 p.S310F, p.R678Q, and p.Q711H.

### RNA expression between HER2 amplified and non-amplified ICCs

**Fig. 5:**
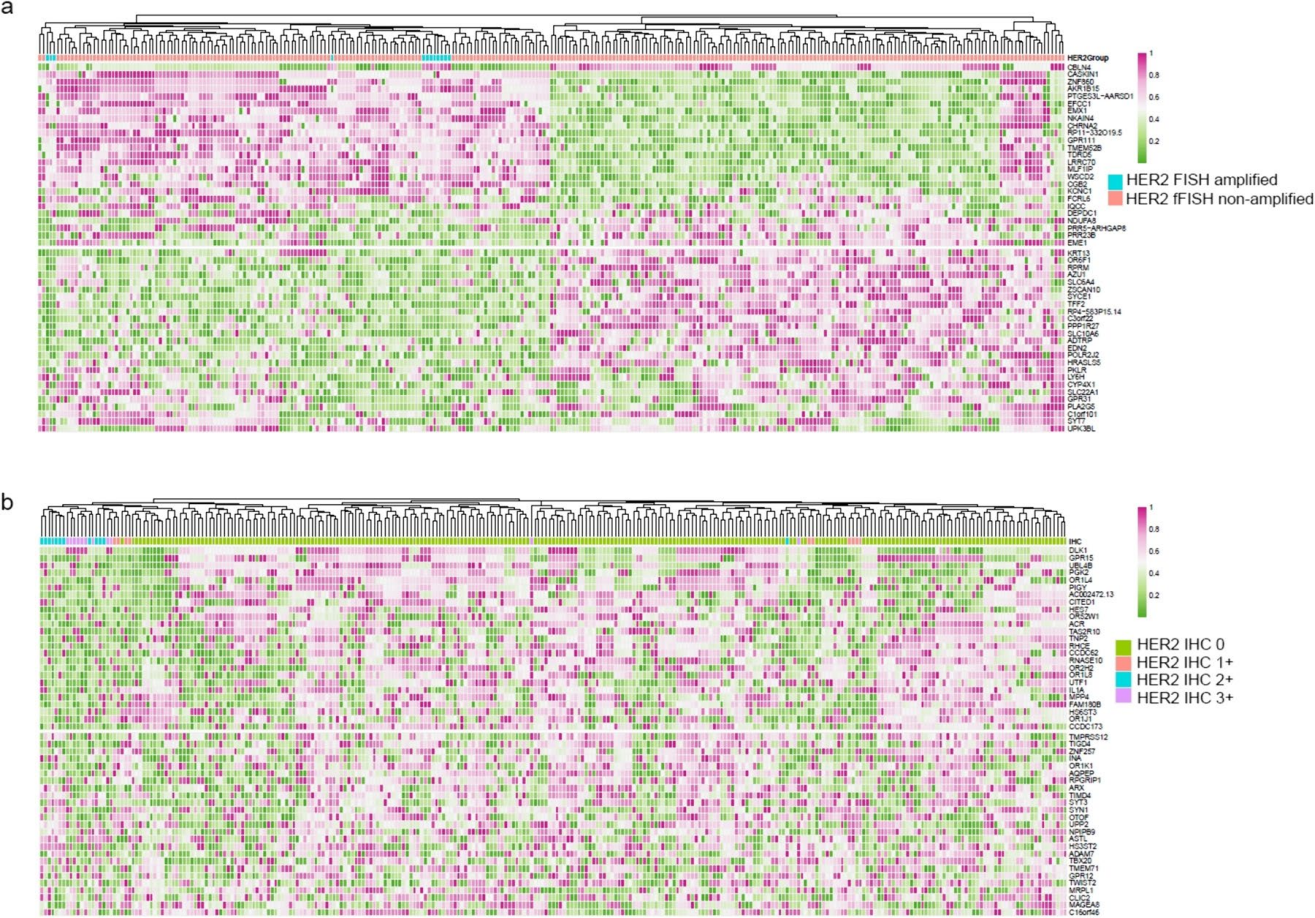
RNA expression between HER2 amplifiedand non-amplified ICCs (**a**), and among different HER2 IHC groups (**b**)

Upon exclusion of tumors with substandard RNA samples, a total of 283 ICCs were available for evaluation of transcriptome expression. Of these, 12 samples displayed HER2 amplification, while the remaining 271 samples exhibited non-amplification of HER2. Based on RNA expression levels, these 283 samples were classified into two clusters. The HER2-amplified subset was categorized into Cluster I, which exhibited high expression of genes such as CBLN4, CASKIN1, ZNF860, and AKR1B15, with concomitant low expression of KRT13, OR6F1, RPRM, and AZU1 (Fig. 5a). Regrettably, no significant divergence in RNA expression levels was discernible between the HER2-amplified and non-amplified groups, preventing the classification of the HER2-amplified group as a distinct subtype based on RNA expression. Although different immunohistochemistry (IHC) groupings also failed to facilitate independent subclassification, cases exhibiting 1 + -3 + in IHC presented similar RNA expression patterns, characterized by lower expression of genes such as PLK1, GPR15, UBL4B, and PGK2 (Fig. 5b).

### Tumor-infiltrating lymphocytes and PD1 expression in HER2 amplification ICC

PD-1/PD-L1, a popular immune checkpoint, is up-regulated in various tumors and induces tumor immune escape (He et al. 2021). Nonetheless, whether the status of HER2 correlate withthe PD1 protein expression and the status of tumor-infiltrating lymphocytes are unknown. Out of the 304 included samples of ICC, a total of 19 samples were subjected to immunophenotypic analysis using whole tumor sections. Among these, 7 samples exhibited HER2 amplification (HER2/CEP17 ≥ 2.0 and HER2 ≥ 4.0; or HER2 ≥ 6.0), while 12 samples with no HER2 amplification were randomly selected as controls. The tumor-infiltrating lymphocytes (TILs) in the central and peripheral regions of the tumors were analyzed separately using markers including CD20, CD3, CD68, CD8, CD4, CD163 and FOXP3. Unfortunately, no significant difference was observed in PD1 and TILs markers expression between the HER2 amplified and non-amplified groups (Supplemental Fig. 2).

### Association between HER2 amplification and TMIT

**Fig. 6:**
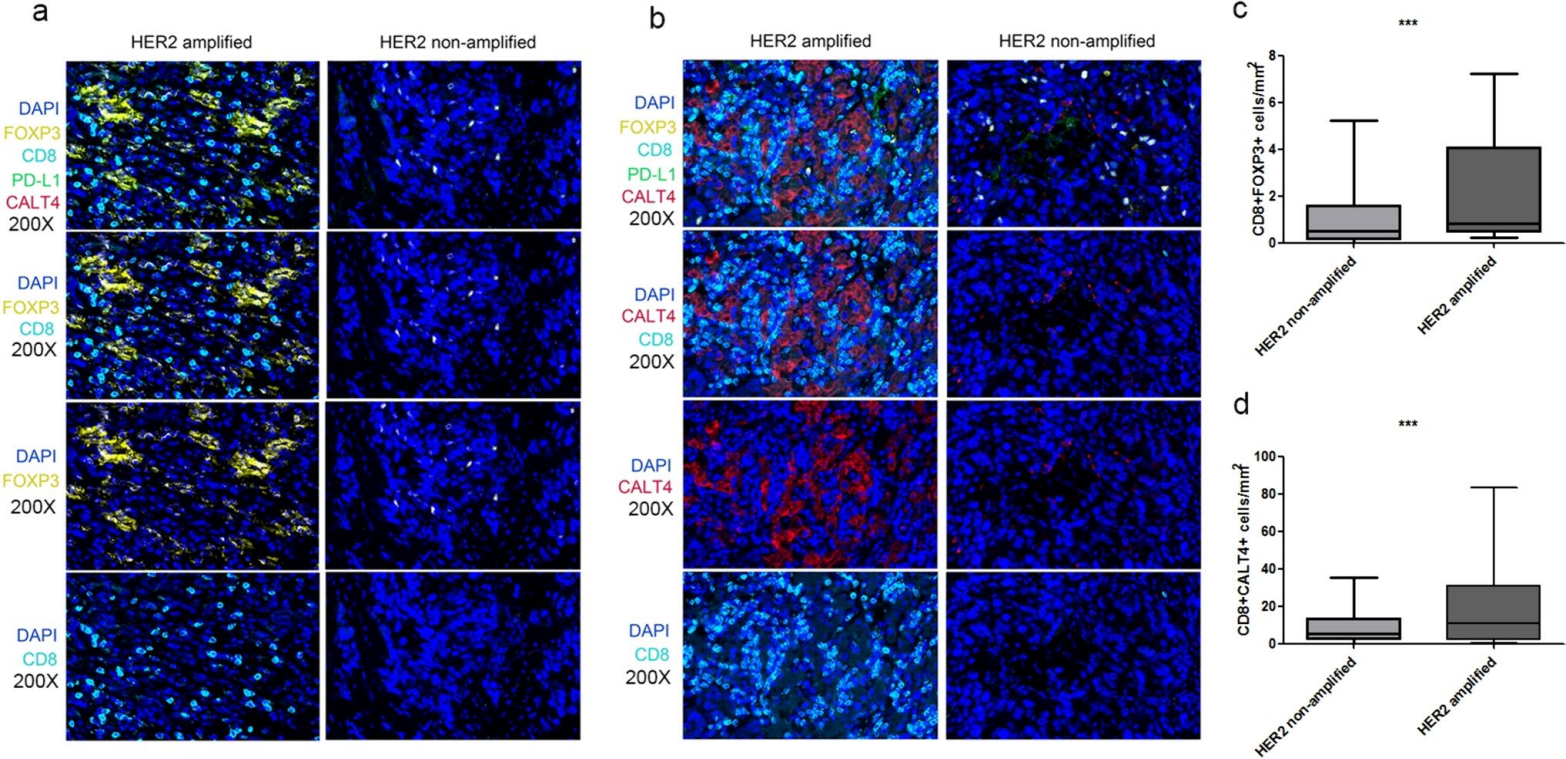
Higher density of CD8 + CTLA4 + (**a** and **c**) and CD8 + FOXP3 + cells (**b** and **d**) in the HER2-amplified group compared with HER2 non-amplified group (all p values < 0.05, based on *t* test)

Despite there being no distinguishable difference in the expression of the PD1 and tumor-infiltrating lymphocyte markers between HER2-amplified and non-amplified ICC groups, the characteristics of their tumor microenvironment remain unexplored. To gain further insights into the tumor microenvironment of HER2-amplified ICC, we conducted multiplex immunofluorescence staining of total tissue slides using a variety of markers. These included two panels: panne1, CD20, CD3, CD68, HER2, and CK in the first panel; and CK, PD-L1, CD8, FOXP3, and CTLA4 in the second panel. Overall, 11 samples which exhibited HER2 amplification and 17 samples with no HER2 amplification were included for multiplex immunofluorescence staining. Confirming our immunohistochemical results, multiplex immunofluorescence also failed to identify notable differences for any single immune marker between the HER2-amplified and non-amplified groups. This was further extended to the density of CD3 + CD8 + cells, indicative of tumor lymphocytic infiltration, which also did not significantly differ between the two groups. However, we observed a higher density of CD8 + CTLA4 + and CD8 + FOXP3 + cells in the HER2-amplified group (Fig. 6). This noteworthy up-regulation suggests that HER2 amplification may elicit a tumor microenvironment encompassing T-cell suppression and immune exhaustion.

## Discussion

The oncogene ERBB2, commonly known as HER2 (human epidermal growth factor receptor 2), is being evaluated as a precision medicine target in biliary tract cancer (Koeberle and Fritsch 2021; Bogenberger et al. 2018). Genomic alterations of ERBB2, including amplifications and mutations, are identified in approximately 20% of biliary tract cancers, with a higher prevalence in extrahepatic cholangiocarcinoma and gallbladder carcinoma, accounting for approximately 15–20% of cases, while the incidence of such alterations in intrahepatic cholangiocarcinoma (ICC) is only 1–5% (Galdy et al. 2017). Despite the significance of HER2 amplification/overexpression as a therapeutic target in various tumor types such as breast cancer, gastric cancer and bladder cancer, HER2's importance in ICC has received limited attention due to its infrequent genomic alterations. In our study, we assessed the genomic status of HER2 using different methodologies and criteria. Based on our established standards, the frequencies of HER2 gene amplification (FISH HER2/CEP17 ≥ 2.0 and HER2 copies ≥ 4, or average HER2 copies ≥ 6), HER2 protein overexpression (IHC 3 +), and HER2 point mutations were identified as 4.28% (13/304), 3. 95% (12/304), and 1.06% (3/283), respectively. All observed mutations were point mutations (HER2 p.S310F, p.R678Q, p.Q711H). Consistent with previous studies, the incidence of HER2 amplification/overexpression in our study cohort was similar to that reported in other studies.

Currently, the detection of HER2 status primarily relies on immunohistochemistry (IHC) and fluorescence in situ hybridization (FISH) techniques, with the criteria for interpretation mainly referencing breast cancer or gastric cancer. However, previous studies on the consistency between HER2 FISH and IHC in biliary tract tumors have yielded inconsistent conclusions. Some studies have shown a strong correlation between IHC expression and amplification (Yang et al. 2014), confirming the reliability of both techniques for HER2 assessment in biliary tract tumors, as already observed in other tumor types (Hofmann et al. 2008; Hanna and Kwok 2006). Conversely, other studies have demonstrated poor consistency, indicating that HER2 gene amplification without HER2 overexpression may still be detected in some biliary tract tumors. In a study focused on the HER2 status in 454 cases of biliary tract tumors, it was observed that a total of 21 samples (4.63%, 21/454) exhibited HER2 gene amplification but lacked protein overexpression (Hiraoka et al. 2020). In another meta-analysis study examining the HER2 status in biliary tract tumors, it was found that the rate of HER2 amplification was approximately 60% among patients exhibiting moderate/strong expression in IHC (Galdy et al. 2017). Our study focused on ICC in the biliary tract tumor, and revealed extremely poor consistency between FISH and IHC, with only 16. 67% of cases with IHC 3 + demonstrating HER2 amplification. This high degree of inconsistency may be attributed to the fact that FISH represents the gene status of HER2, while IHC reflects the protein status, which undergoes complex regulation during the process from gene expression to protein expression. Additionally, the evaluation criteria for gastric and breast cancer may not be suitable for ICC. In clinical practice, the HER2 expression status is often determined by surgical resection sample evaluation. However, intratumoral heterogeneity is apotential cause of false negative results in the evaluation basedon small samples (Allison et al. 2011; Nitta et al. 2016). In this study, we observed high intratumoral heterogeneity in IHC, with 7.57% of samples exhibiting inconsistent results from two tests. In contrast, FISH exhibited much lower intratumoral heterogeneity, with only 1.98% of samples showing inconsistent results from the two TME scores. Moreover, previous studies in breast and gastric cancer have suggested thatregional intratumoral heterogeneity affects response to anti-HER2therapies (Yagi et al. 2019; Filho et al. 2021; Lee et al. 2015). This suggests that FISH is a more acceptable method for the determination of HER2 status in ICC. Moreover, in clinopathological features, we discovered that cases with HER2 gene amplification (FISH HER2/CEP17 ≥ 2.0 and HER2 copies ≥ 4, orHER2/CEP17 < 2.0 and average HER2 copies ≥ 6) exhibited poorer prognosis. However, neither the gene status nor protein status of HER2 could differentiate the clinical and pathological differences in ICC, including age, gender, etiology, tumor size, differentiation grade, and G/S/T staging. Given the influence of various factors such as the distinct classifications of FISH and IHC in differentiating prognosis, clinical and pathological characteristics, and tumor heterogeneity in outcomes, we recognize the potential of detecting the gene status of HER2 as being more effective than evaluating its protein status.

There is currently no description of HER2 gene amplification/overexpression subtype mutation characteristics in cholangiocarcinoma; however, research in colorectal cancer has found that all HER2-amplified tumors lacked activating mutations of MAPK pathway genes (including KRAS, NRAS, and BRAF) except for two tumors with KRAS mutations. In contrast, half of the HER2-low tumors carried activating RAS/BRAF mutations. Meanwhile, the frequencies of PIK3CA mutation in HER2-amplified and HER2-low expression tumors were not significantly different from those in HER2-negative tumors. All HER2-amplified tumors were MMR-proficient, whereas 13% of HER2-low expression tumors were MMR-deficient in colorectal cancer (Hashimoto et al. 2023). Our results also found that the frequency of KRAS mutations in the HER2-amplified group was lower than in the non-amplified group.Among the 12 HER2-amplified samples tested by NGS, two had KRAS gene mutations (17%), while the frequency of KRAS mutations in the 271 non-amplified samples was 25%. However, due to the extremely low frequency of mismatch repair protein deficiency in ICC, only 2 of the total 304 samples were found to have MLH2/PMS2 deficiency, both of which were HER2 non-amplified cases. We have not yet discovered any correlation between HER2 gene status and mismatch repair protein expression. Nonetheless, we observed that the tumor mutation burden in the HER2-amplified ICC subtype was significantly higher than in the non-amplified group.

The study of the Tumor Immune Microenvironment (TIME) in ICC, particularly with respect to varying HER2 status, remains an underexplored field. Certain previous studies, however, have managed to shed light on the immune characteristics of distinctive genotypic subtypes of ICC. For instance, IDH mutant subgroupe exhibits less T cell infiltration and lower T cell cytotoxicity, indicating a colder tumor microenvironment (TME) (Xiang et al. 2021). In our research, we detected an increased density of CD8 + CTLA4 + and CD8 + FOXP3 + cells within the HER2-amplified group.Cytotoxic T-Lymphocyte Associated protein 4 (CTLA4) serves as a co-inhibitory molecule that becomes active upon expression on the CD4 + and CD8 + T lymphocytes. With a higher affinity for the ligands CD80 and CD86 on antigen-presenting cells (APCs) than the co-stimulation receptor CD28 on T cells, the interaction between CTLA4 and its ligands suppresses T-cell activity, thereby promoting tumor growth. As a result, CTLA4 is viewed as a physiological "brake" triggered by APC on CD4 + and CD8 + T cell activation. Inhibition of the interaction between CTLA4 and its ligands allows T cells to remain active and target and destroy tumor cells (Park et al. 2016; Camisaschi et al. 2016). Regulatory T cells (Tregs) encompass a group of T cells exhibiting immune suppressive properties. These cells are further divided into natural Tregs (CD4 + CD25 + Treg) and induced Tregs (iTreg), including CD4 + Treg and CD8 + Treg types. The latter involves numerous subcategories, including CD8 + CD122 + Treg, CD8 + CD28-Treg, CD8 + CD103 + Treg, CD8 + FOXP3 + Tregs (Stockis et al. 2019). In this context, FOXP3 plays an integral role in the development and differentiation of Tregs and in mediating tumor immune evasion, making it an effective target for identifying Tregs in the tumor microenvironment. A preventative FOXP3 DNA ecombinant protein vaccine can promote immunity against Tregs in the absence of a tumor, enhancing the immune response against the tumor by targeting Tregs and Myeloid-derived suppressor cells (MDSCs). This method has potential as an immunotherapeutic approach (Namdar et al. 2018). Thus, employing immunosuppressants to inhibit the expression of CTLA4 or FOXP3 may emerge as a potential treatment strategy for HER2-amplified subtypes of ICC. Furthermore, distinct research findings suggest that the immune response is closely linked to patient prognostic outcomes and the effectiveness of immunotherapeutic interventions, underscoring the need for a comprehensive examination of tumor microenvironment immune escape (TMIE) in ICC. Nevertheless, it's important to acknowledge the significant variability in the extent and composition of TMIE within individual instances of HER2 amplified ICC.

In summary, we defined fluorescent in situ Hybridization (FISH) positive criteria as HER2/CEP17 ≥ 2.0 and HER2 ≥ 4.0 or HER2 ≥ 6.0, therefore collecting a total of 13 (4.27%) HER2-amplified samples during this investigation. Of the 304 samples analyzed, 7.57% (23/304) demonstrated inconsistencies in Immunohistochemistry (IHC) findings, whereas 1.97% (6/304) exhibited heterogeneity with FISH. The agreement between IHC and FISH for verification was merely 44.07%, asserting FISH as the gold standard for determining HER2 amplification in ICC. Our insights indicate the HER2 genetic status as a determinant for ICC prognosis. However, a combination of HER2 genetic and protein statuses were ineffective in distinguishing clinical pathological features of ICC. Relative to non-amplified cases, HER2-amplified counterparts manifested a higher tumor mutational burden. Nevertheless, no significant discrepancies were discerned in immune markers between the groups. Interestingly, our study identified an escalated density of CD8 + CTLA4 + and CD8 + FOXP3 + cells in HER2 gene-amplified instances. However, it's crucial to acknowledge that this study did not accomplish the evaluation of tumor-infiltrating lymphocytes and PD-L1 expression, as well as the tumor immune microenvironment assessment in the entirety of the samples, which presents a limitation of this research.

## Supplementary Information

Below is the link to the electronic supplementary material.

Supplemental Figure 1 Kaplan-Meier curves of overall survival among FISH subgroups. HER2/CEP17<2.0 and HER2<4.0 (HER2 non-amplified) vs. HER2/CEP17≥2.0 and HER2≥4.0, or HER2≥6.0; 5.0≤HER2<6.0; 4.0≤HER2<6.0 (a).Kaplan-Meier curves of overall survival among IHC subgroups, IHC 0 vs. IHC 1+(b), IHC 0 vs. IHC 2+(c) and IHC 0 vs. IHC 3+(d). p value relates to the log rank analysis and median survival in months for each group was presented in the table (JPG 242 KB)

Supplemental Figure 2 PD1 and TILs markers (CD20, CD3, CD68, CD8, CD4, CD163 and FOXP3) expression between the HER2 amplified and non-amplified groups. The tumor-infiltrating lymphocytes (TILs) in the centralmass (CM) and peripheral areas(PA) of the tumors were analyzed separately (JPG 491 KB)

Supplementary file3 (DOC 91 KB)

## Data Availability

All data have been uploaded as supplementary data and can be downloaded if necessary.
