# Supplementary figures and images for: HER2 amplification subtype intrahepatic cholangiocarcinoma exhibits high mutation burden and T cell exhaustion microenvironment
Source: J Cancer Res Clin Oncol. 2024 Aug 28;150(8):403. doi: 10.1007/s00432-024-05894-0 (PMC11358322; doi:10.1007/s00432-024-05894-0)

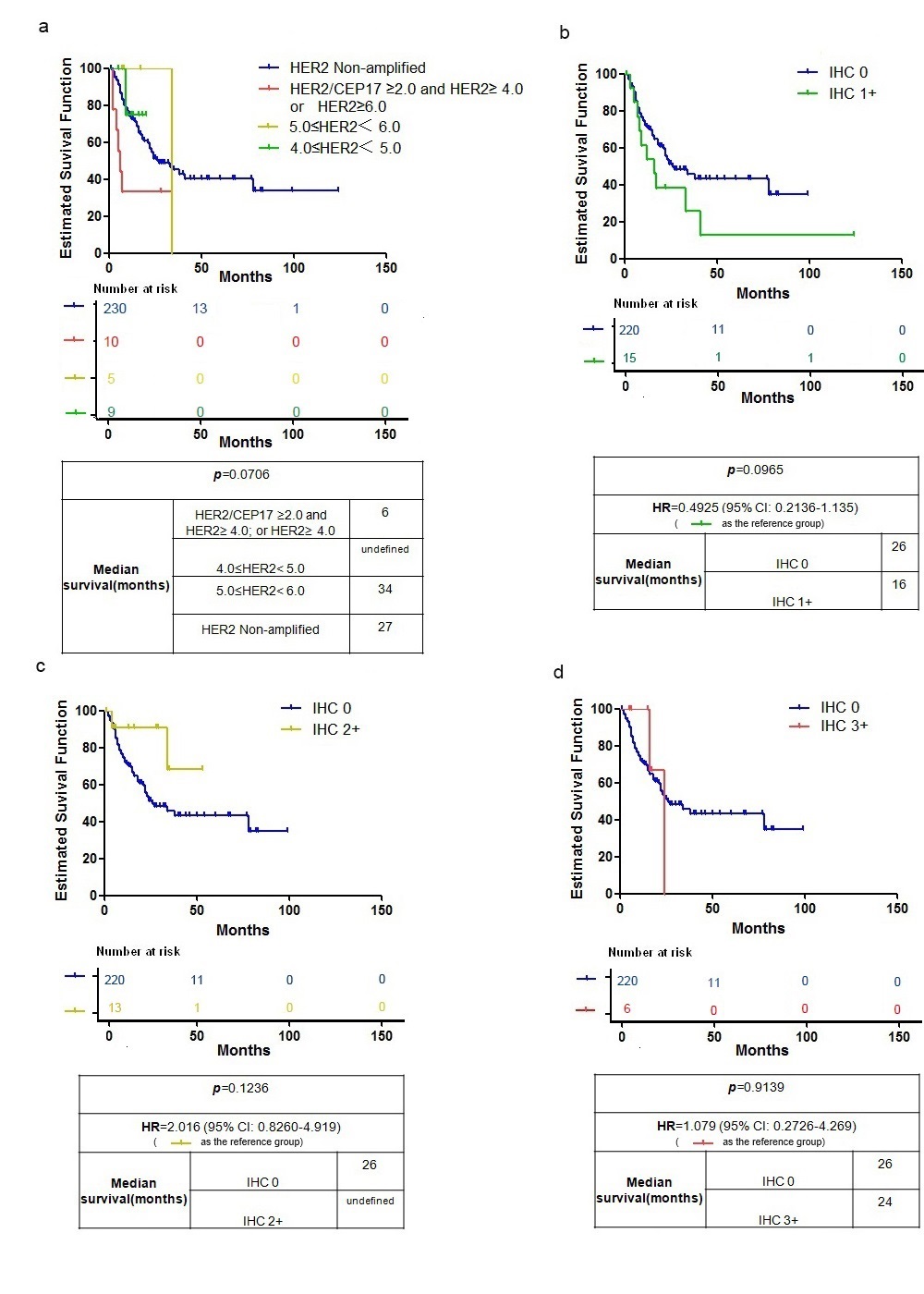

Supplement: Supplementary file 1 — Supplemental Figure 1 Kaplan-Meier curves of overall survival among FISH subgroups. HER2/CEP17<2.0 and HER2<4.0 (HER2 non-amplified) vs. HER2/CEP17≥2.0 and HER2≥4.0, or HER2≥6.0; 5.0≤HER2<6.0; 4.0≤HER2<6.0 (a).Kaplan-Meier curves of overall survival among IHC subgroups, IHC 0 vs. IHC 1+(b), IHC 0 vs. IHC 2+(c) and IHC 0 vs. IHC 3+(d). p value relates to the log rank analysis and median survival in months for each group was presented in the table (JPG 242 KB) [file 432_2024_5894_MOESM1_ESM.jpg]

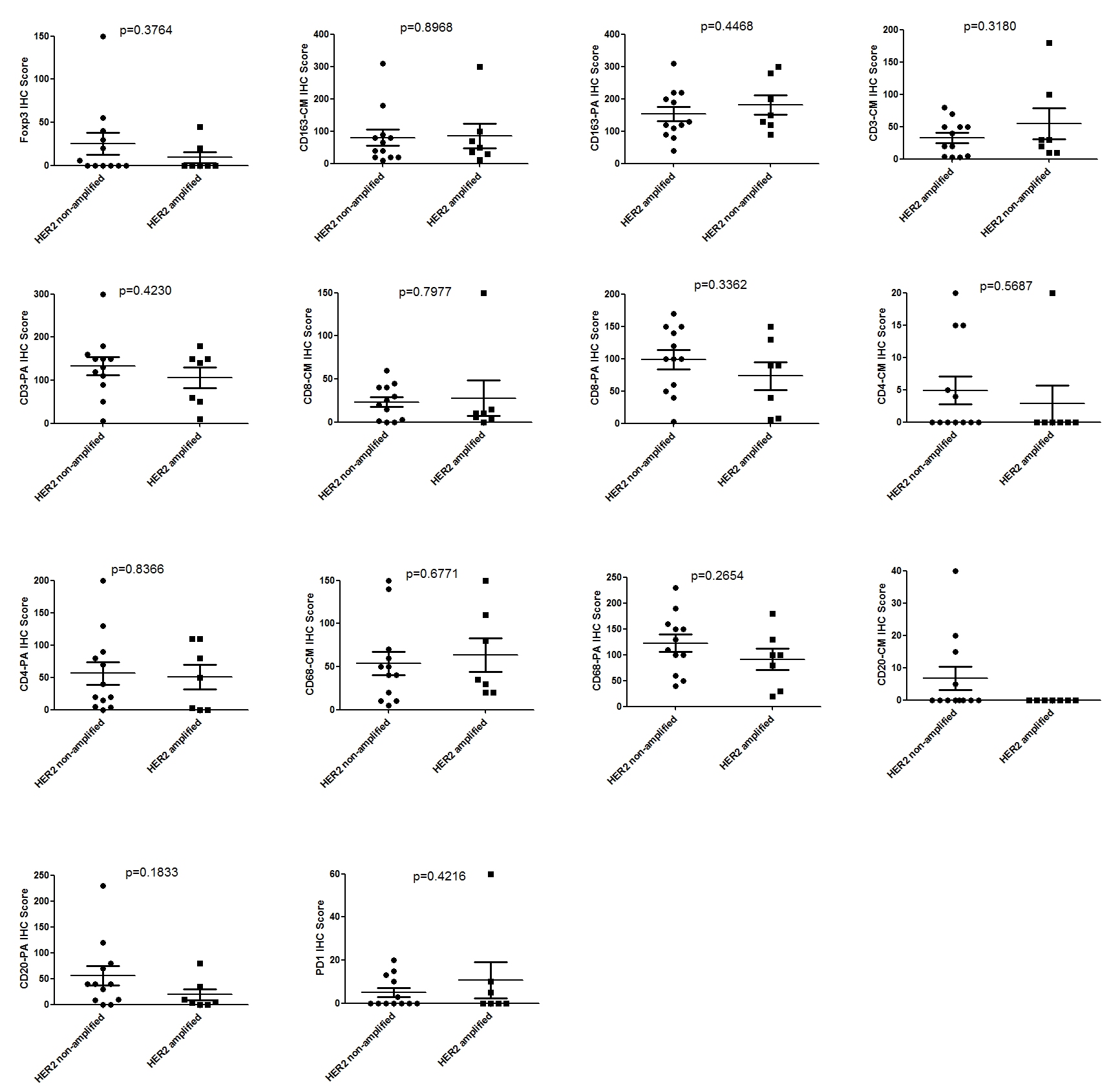

Supplement: Supplementary file 2 — Supplemental Figure 2 PD1 and TILs markers (CD20, CD3, CD68, CD8, CD4, CD163 and FOXP3) expression between the HER2 amplified and non-amplified groups. The tumor-infiltrating lymphocytes (TILs) in the centralmass (CM) and peripheral areas(PA) of the tumors were analyzed separately (JPG 491 KB) [file 432_2024_5894_MOESM2_ESM.jpg]
