# Supplementary material for: HER2 amplification subtype intrahepatic cholangiocarcinoma exhibits high mutation burden and T cell exhaustion microenvironment
Source: J Cancer Res Clin Oncol. 2024 Aug 28;150(8):403. doi: 10.1007/s00432-024-05894-0 (PMC11358322; doi:10.1007/s00432-024-05894-0)
Supplement: Supplementary file 3 — Supplementary file3 (DOC 91 KB) [file 432_2024_5894_MOESM3_ESM.doc]

Supplemental table 1 Clinicopathologic characteristics of HER2 amplification and non-amplification in ICCs

| No. Patients | HER2 amplification  (N=13) | HER2 non-amplification (N=291) | *p* value |
| --- | --- | --- | --- |
| Average age (year)mean(min-max)  Gender  Male  Female | 64.85（52-76）  7(53.8%)  6(46.2%) | 60.84（34-88）  146/145(50.2%/49.8%) | 0.158  1.000 |
| HBV infection  Yes  No  Dipsomania  Yes  No  Biliary hamartoma  Yes  No  Clonorchiasis  Yes  No | 5(38.5%)  8(61.5%)  2(15.4%)  11(84.6%)  1(7.7%)  12(92.3%)  0(0)  13(100%) | 121 (41.6%)  170 (58.4%)  25(8.6%)  266(91.4%)  18(6.2%)  273(93.8%)  5(1.7%)  286(98.3%) | 1.000  0.324  0.575  1.000 |
| Tumor numbers(n)  Tumormaximum dimension (cm) | 1.14  5.71 | 1.31  6.90 | 0.093  0.487 |
| Histological classification  Large duct  Small duct  Cholangiolocarcinoma | 2(15.4%)  7(53.8%)  4(30.8%) | 62(21.3%)  147(50.5%)  82(28.2%) | 0.876 |
| mVI  0  1  2 | 11(84.6%)  2(15.4%)  0(0) | 210(72.2%)  80(27.5%)  1(0.3%) | 0.611 |
| Differentiation  L  M  H  U | 0(0)  4(30.8%)  9(69.2%)  0(0) | 4/(1.4%)  135(46.4%)  150(78.5%)  2(0.6%) | 0.642 |
| G  0  1  2  3  4  S  0  1  2  3  4  T  I  II  III  IV  Stage  I  II  III  IV | 0(0)  9(69.2%)  3(23.1%)  1(7.7%)  0(0)  1(7.7%)  9(69.2%)  0(0)  1(7.7%)  2(15.4%)  7(53.8%)  4(30.8%)  2(15.4%)  0(0)  5(38.5%)  2(15.4%)  5(38.5%)  1(7.8%) | 24(8.3%)  140(48.1%)  91(31.3%)  33(11.3%)  3(1.0%)  96(32.9%)  107(36.8%)  47(16.2%)  14(4.8%)  27(9.3%)  150(51.5%)  96(33.0%)  41(14.1%)  4(1.4%)  119(40.9%)  80(27.5%)  68(23.3%)  24(8.3%) | 0.599  0.068  0.973  0.598 |
| Ki67 | 30.32 | 40.38 | 0.072 |

Abbreviations: L, low differentiated; M, moderately differentiated; H, high differentiated; U, undifferentiated; G, grade; S, stage; T, tumor.

Supplemental table 2 Clinicopathologic characteristics of HER2 IHC 0, 1+, 2+, 3+ in ICCs

| No. Patients | IHC 0 (N=260) | IHC 1+ (N=18) | IHC2+ (N=14) | IHC3+(N=12) | *p* value |
| --- | --- | --- | --- | --- | --- |
| Average age (year)  Gender  Male  Female | 60.98  133(51.2%)  127(48.2%) | 60.11  8(44.4%)  10(55.6%) | 60.93  8(57.1%)  6(42.9%) | 63.17  4(33.3%)  8(66.7%) | 0.579 |
| HBV infection  No  Yes  Dipsomania  Yes  No  Biliary hamartoma  Yes  No  Clonorchiasis  No  Yes | 149(57.3%)  111(42.7%)  236(90.8%)  24(9.2%)  246(94.6%)  14(5.4%)  255(98.1%)  5(1.9%) | 13(72.2%)  5(27.8%)  17(94.4%)  1(5.6%)  16(88.9%)  2(11.1%)  18(100%)  0(0) | 9(64.3%)  5(35.7%)  13(92.9%)  1(7.1%)  12(85.7%)  2(14.3%)  14(100%)  0(0) | 7(58.3%)  5(41.7%)  11(91.7%)  1(8.3%)  11(91.7%)  1(8.3%)  12(100%)  0(0) | 0.628  0.952  0.442  0.835 |
| Tumor numbers(n)  Tumor maximum dimension (cm) | 1.14  5.69 | 1.29  6.14 | 1.14  7.00 | 1.00  5.15 |  |
| Histological classification  Large duct  Small duct  Cholangiolocarcinoma | 52  135  73 | 5  8  5 | 5  5  4 | 2  6  4 | 0.807 |
| mVI  0  1  2 | 190(73.1%)  69(26.5%)  1(0.4%) | 11(61.1%)  7(38.9%)  0(0) | 10(71.4%)  4(28.6%)  0(0) | 10(83.3%)  2(16.7%)  0(0) | 0.906 |
| Differentiation  L  M  H  U | 4(1.5%)  114(43.8%)  140(153.8%)  2(0.9%) | 0(0)  11(61.1%)  7(38.9%)  0(0) | 0(0)  6(42.9%)  8(57.1%)  0(0) | 0(0)  8(66.7%)  4(33.3%)  0(0) | 0.839 |
| G  0  1  2  3  4  S  0  1  2  3  4  T  I  II  III  IV  Stage  I  II  III  IV | 20(7.7%)  130(50.0%)  80(30.8%)  28(10.7%)  2(0.8%)  82(31.5%)  93(35.8%)  43(16.5%)  13(5.0%)  29(311.2%)  130(50.0%)  89(34.2%)  38(14.6%)  3(1.2%)  104(40.0%)  76(29.2%)  62(23.9%)  18(6.9%) | 3(16.7%)  7(38.9%)  6(33.3%)  1(5.6%)  1(5.6%)  5(27.7%)  12(66.7%)  0(0)  1(5.6%)  0(0)  8(44.4%)  8(44.4%)  2(11.2%)  0(0)  5(27.8%)  3(16.7%)  4(22.2%)  6(33.3%) | 1(7.2%)  8(57.1%)  3(21.4%)  2(14.3%)  0(0)  4(28.6%)  8(57.1%)  1(7.1%)  1(7.1%)  0(0)  10(71.5%)  3(21.4%)  1(7.1%)  0(0)  8(57.2%)  3(21.4%)  2(14.3%)  1(7.1%) | 0(0)  4(33.3%)  5(41.7%)  3(25.0%)  0(0)  6(50.0%)  3(25.0%)  3(25.0%)  0(0)  0(0)  9(75.0%)  0(0)  2(16.7%)  1(8.3%)  7(58.3%)  0(0)  5(41.7%)  0(0) | 0.467  0.134  0.134  0.003 |
| Ki67 | 31.06+20.102 | 28.24+17.495 | 28.15+16.886 | 30.80+18.273 |  |

Supplemental table 3 Comparison between IHC and FISH results.

| HER2 IHC Value | Number | HER2 FISH positive Number |
| --- | --- | --- |
| 0 | 260 | 8 |
| 1+ | 18 | 0 |
| 2+ | 14 | 3 |
| 3+ | 12 | 2 |
| overall | 304 | 13 |

Supplemental table 4 Cases with heterogeneity in FISH results.

| Sample No. | Core 1 FISH result | | | Core 2 FISH result | | | Whole slide FISH result | | | Final result |
| --- | --- | --- | --- | --- | --- | --- | --- | --- | --- | --- |
|  | HER2/CEP17 | HER2 | CEP17 | HER2/CEP17 | HER2 | CEP17 | HER2/CEP17 | HER2 | CEP17 |  |
| 1 | 1.36 | 6.10 | 4.50 | 1.11 | 4.20 | 3.80 | 1.25 | 4.55 | 3.65 | Non-amplified |
| 2 | 2.98 | 6.40 | 2.15 | 1.55 | 3.50 | 2.25 | 2.82 | 6.20 | 2.20 | Amplified |
| 3 | 2.21 | 4.65 | 2.10 | 1.95 | 3.90 | 2.00 | 1.88 | 3.95 | 2.1 | Non-amplified |
| 4 | 2.20 | 6.05 | 2.75 | 1.95 | 5.85 | 3.0 | 2.39 | 6.10 | 2.55 | Amplified |
| 5 | 2.53 | 4.80 | 1.90 | 1.78 | 4.55 | 2.55 | 1.76 | 4.50 | 2.55 | Non-amplified |
| 6 | 1.67 | 6.10 | 3.65 | 1.21 | 4.85 | 4.00 | 1.34 | 4.90 | 3.65 | Non-amplified |
